# DEPRESSION AND LONELINESS IN BLACK WOMEN WITH HYPERTENSION: A SYSTEMATIC LITERATURE REVIEW

**DOI:** 10.1093/geroni/igad104.2435

**Published:** 2023-12-21

**Authors:** Lashawn Hutto, Michelle Odlum, Maritza Dowling, Laurie Theeke

**Affiliations:** George Washington University, Washington, District of Columbia, United States; The George Washington University, Washington, District of Columbia, United States; George Washington University, Washington, District of COlumbia, United States; The George Washington University, Washington, District of Columbia, United States

## Abstract

Hypertension occurs more frequently in Black women than men. Over 50% of Black women have hypertension, compared to 39% of non-Hispanic white women and 38% of Hispanic women. Black women with hypertension are more likely to report depressive symptoms which are associated with hypertension, a known risk for cardiovascular diseases including myocardial infarction, heart failure, and stroke. This paper presents a synthesis of findings from quantitative studies that measured depression and loneliness in black women with hypertension. The literature search used EbscoHost with the following databases: Medline Complete, Academic Search Complete, Medline, Pub-Med, Scopus, APA PsycArticles, APA PsycINFO, and CINAHL. Twelve articles were included. Loneliness and social isolation are predictive of depression in black women ages 40 and older. The issue is further confounded by cultural norms that include not seeking therapy, discussing symptoms, and displaying vulnerability, leading to loneliness and disconnection. Loneliness is increased in middle age black women who’ve experienced traumatic events. In an intervention trial, the associations between blood pressure control and living arrangements were examined. Middle- aged black women living alone were reported to have a higher prevalence of uncontrolled blood pressure, cardiovascular events, and mortality compared to those living with others. There are limited studies of loneliness and depression in black women with hypertension. Additional studies are needed that include variables reflective of cultural norms. A culturally sensitive clinical environment that facilitates discussion about feelings of depression and loneliness could be foundational to the development of new more precise interventions for black women with hypertension.

